# Immune cells and their related genes provide a new perspective on the common pathogenesis of ankylosing spondylitis and inflammatory bowel diseases

**DOI:** 10.3389/fimmu.2023.1137523

**Published:** 2023-03-30

**Authors:** Yimei Ding, Yue Yang, Luan Xue

**Affiliations:** Department of Rheumatology and Immunology, Yueyang Hospital of Integrated Traditional Chinese and Western Medicine, Shanghai University of Traditional Chinese Medicine, Shanghai, China

**Keywords:** ankylosing spondylitis, inflammatory bowel diseases, neutrophil, T-cell, pathogenesis

## Abstract

**Background:**

The close relationship between ankylosing spondylitis (AS) and inflammatory bowel diseases (IBD) has been supported by many aspects, including but not limited to clinical manifestations, epidemiology and pathogenesis. Some evidence suggests that immune cells actively participated in the pathogenesis of both diseases. However, information on which cells are primarily involved in this process and how these cells mobilize, migrate and interact is still limited.

**Methods:**

Datasets were downloaded from Gene Expression Omnibus (GEO) database. Common differentially expressed genes (coDEGs) were identified by package “limma”. The protein-protein interaction (PPI) network and Weighted Gene Co-Expression Network Analysis (WGCNA) were used to analyze the interactions between coDEGs. KEGG pathway enrichment analysis and inverse cumulative distribution function were applied to identify common differential pathways, while Gene Set Enrichment Analysis (GSEA) was used to confirm the significance. Correlation analysis between coDEGs and immune cells led to the identification of critical immune-cell-related coDEGs. The diagnostic models were established based on least absolute shrinkage and selection operator (LASSO) regression, while receiver operating characteristic (ROC) analysis was used to identify the ability of the model. Validation datasets were imported to demonstrate the significant association of coDEGs with specific immune cells and the capabilities of the diagnostic model.

**Results:**

In total, 67 genes were up-regulated and 185 genes were down-regulated in both diseases. Four down-regulated pathways and four up-regulated pathways were considered important. Up-regulated coDEGs were firmly associated with neutrophils, while down-regulated genes were significantly associated with CD8+ T−cells and CD4+ T−cells in both AS and IBD datasets. Five up-regulated and six down-regulated key immue-cell-related coDEGs were identified. Diagnostic models based on key immue-cell-related coDEGs were established and tested. Validation datasets confirmed the significance of the correlation between coDEGs and specific immune cells.

**Conclusion:**

This study provides fresh insights into the co-pathogenesis of AS and IBD. It is proposed that neutrophils and T cells may be actively involved in this process, however, in opposite ways. The immue-cell-related coDEGs, revealed in this study, may be relevant to their regulation, although relevant research is still lacking.

## 1 Introduction

Both ankylosing spondylitis (AS) and inflammatory bowel diseases (IBD) are chronic relapsing inflammatory disorders involving the joints and intestines, respectively (1, 2). The close relationship between AS and IBD has been supported by various aspects, including but not limited to clinical manifestations, epidemiology and pathogenesis. IBD is one of the most common extra-articular features of AS while arthritis and spondyloarthropathy are classical extra-intestinal manifestations of IBD (3). Moreover, inflammatory bowel diseases arthritis (IBDA) together with ankylosing spondylitis belong to a broader category known as spondyloarthritis (SpA), indicating the semblable manifestations. The prevalence of IBD ranges from 6%-14% in patients with AS (4). Meanwhile, AS is encountered in about 3%(95% CI 2-4%) of patients with IBD (5).

In addition to clinical research, studies on genes, molecules and cells provide extra information for the correlation between AS and IBD. Some disease-related genes are shared between AS and IBD, of which IL23R is a typical representative (6), though, IL23A has been found associated with IBD but not with AS (7). The intestinal barrier is thought to play an essential role in the pathogenesis of AS, and thus a hypothesis focusing on the inflammation of gut has been proposed. “Gut-synovial axis” hypothesis, implicating environmental and host factors, tries to establish a connection between the intestine and the joint (4). On one hand, intestinal dysbiosis and the disruption of epithelial barrier initially trigger the activation of innate cells, innate-like cells and some subsets of T cells in SpA (8). On the other hand, microscopic gut inflammation in patients with AS is considered to be an early stage of Crohn’s disease (9).

Immune migration initiated from the gut has also been supported by several experiments. For example, CX3CR1+ mononuclear phagocytes (MNP), considered to be tissue-resident, could acquire migratory potential and move to secondary lymphoid organs during intestinal inflammation (10). CX3CR1+CD59+ MNPs expanded not only in inflamed gut tissue but also in the peripheral blood, synovial fluid, synovial tissue and bone marrow of patients with AS. The expression of CCR9, a marker of intestinal homing, in non-intestinal CX3CR1+CD59+ cells probably indicated their intestinal origin (11, 12). Type 3 innate lymphoid cells (ILC3), a type of mucosal-restricted lymphoid cell, expanded in the blood, bone marrow and synovial fluid in patients with AS and the blood, synovial and bone marrow ILC3s expressed the intestinal homing molecule α4β7 integrin (13). From another perspective, the genetic background of AS adversely affects intestinal balance. HLA-B27 influenced the gut microbiome composition in AS patients (14). HLA-B27 transgenic rats also exhibited microbiota-dependent intestinal inflammation with the Lin-CD172a+CD43low monocytes expansion (15).

It is widely accepted that both innate and adaptive immunity participate in the process, while growing evidence supports a higher dominant position of innate immunity. Innate cells, including mast cells, neutrophils and macrophages, as well as innate lymphoid cells and innate-like T cells were reported to be involved in the pathogenesis of AS (8). However, although there have been some experiments that offer possible explanations, our understanding of the mechanism is still just the tip of the iceberg. Hitherto, the information about how these cells mobilize, migrate and interact with each other is still limited. The study focused on the common differentially expressed genes (coDEGs) in AS and IBD patients’ peripheral blood which communicate the intestines and joints. Further exploration concentrated on the mutual mechanism, especially the relationship between coDEGs and immune cells. The results provide new insights into the pathogenesis of AS and IBD, and even more, add some building blocks to the bridge of gut and joint.

## 2 Materials and methods

**Figure 1:**
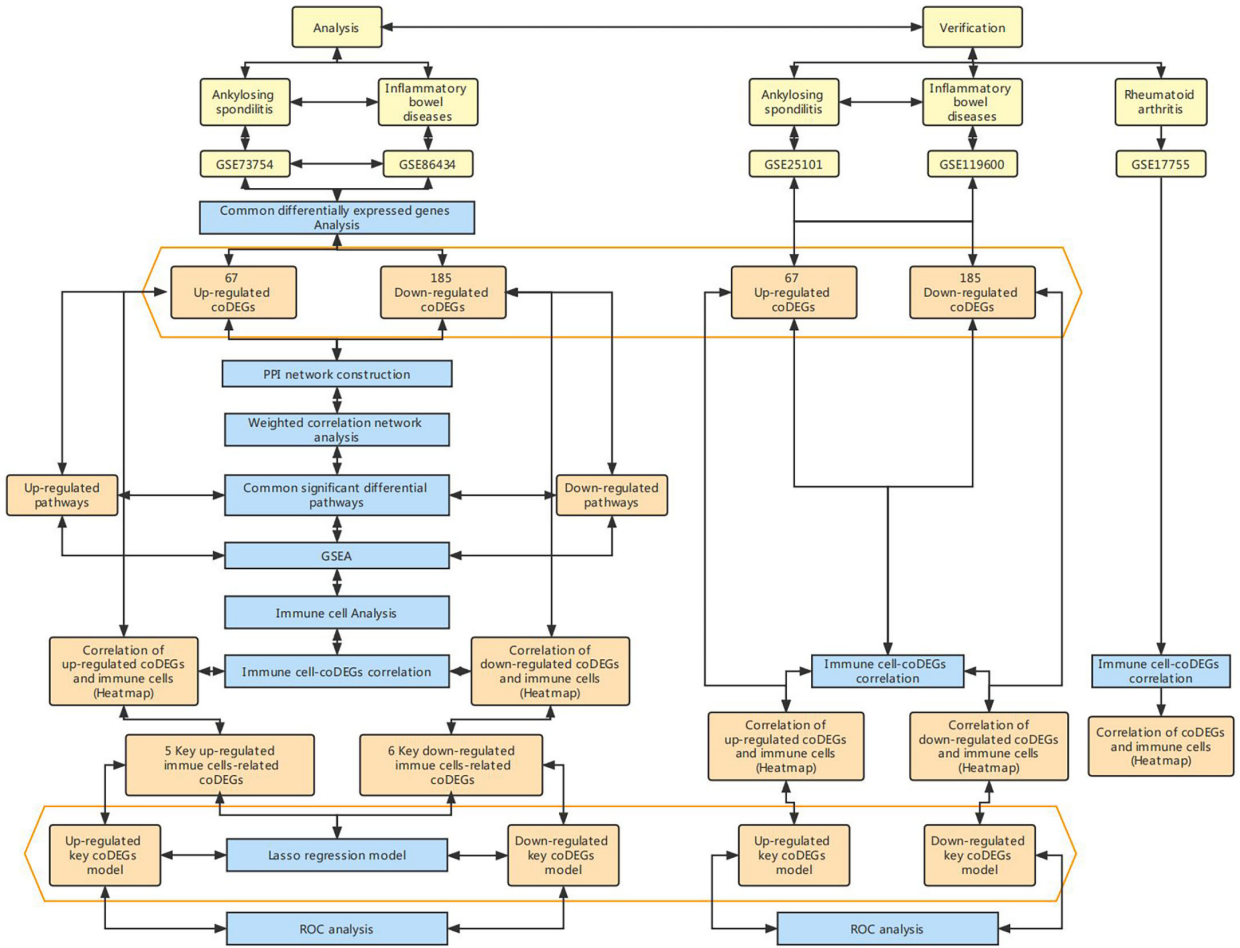
Flow chart of research design.

The overall design of the study was presented in **Figure 1**.

### 2.1 Datasets

Gene expressions were obtained from Gene Expression Omnibus (GEO) database (http://www.ncbi.nlm.nih.gov/geo/) with the type of microarray. Search terms were “ankylosing spondylitis” and “inflammatory bowel disease” while organism and tissue were limited to homo sapiens and blood respectively. Two datasets of AS (GSE73754 and GSE25101) and two datasets of IBD (GSE86434 and GSE94648) were finally included in the study. GSE73754 and GSE86434 were used for analysis and model training, while GSE25101 and GSE94648 were used for model testing. An additional dataset of rheumatoid arthritis (RA), GSE17755, was used for reverse validation. Data were normalized with the function “normalizeBetweenArrays” in package “limma”.

### 2.2 Identification of common differentially expressed genes

Function “Lmfit” in package “limma” was used to fit linear model for each gene in the given series of arrays. Function “contrasts.fit” was used to compute estimated coefficients and standard errors. Genes with P-value < 0.05 were considered to be differentially expressed genes. Genes that were up-regulated or down-regulated in both AS and IBD were defined as common differentially expressed genes (coDEGs). Volcano plots were generated using package “ggplot” to display the differentially expressed genes in AS and IBD. Venn plots were done with package “ggVennDiagram” to show the coDEGs between AS and IBD.

### 2.3 The protein-protein interaction network construction

The protein-protein interaction (PPI) network was analyzed according to STRING Database (the Search Tool for the Retrieval of Interacting Genes, http://string-db.org). The interactions included direct (physical) and indirect (functional) associations.

### 2.4 Weighted correlation network analysis

Weighted correlation network analysis (WGCNA) was imported to find clusters (modules) of greatly correlated coDEGs using package “WGCNA”. Genes in the same module were considered more likely to be regulated together.

### 2.5 Identification of significant differential pathways with inverse cumulative distribution function

Package “clusterProfiler” was used to perform KEGG pathway enrichment analysis of coDEGs. Significantly enriched KEGG terms were screened with adjust P-value < 0.05. On the basis of KEGG enrichment analysis, the degree to which a certain pathway deviated from normal was further analyzed. The P-value, standing for significance of each gene, was converted to Z-score by inverse cumulative distribution function. Adjusted score was calculated based on the Z-score of all the differential expressed genes in this pathway. The adjusted score was displayed in color in the diagram of KEGG analysis, with darker colors indicating more distinct pathways. Package “ggplot2” was used to display the results.

### 2.6 Identification of common differential pathways and gene set enrichment analysis

The pathways that were up-regulated or down-regulated in both AS and IBD were considered as possible co-acting pathways. Both gene counts and adjusted scores in enrichment analysis were considered to determine the common differential pathway. Since the KEGG enrichment analysis was based on coDEGs, further analysis was necessary to determine whether these pathways were significant in the overall biological state. Thus, gene set enrichment analysis (GSEA) was applied to the common differential pathways to clarify whether the pathway was significantly different between patients with AS or IBD and healthy controls. GSEA was applied to the gene sets of AS and IBD separately.

### 2.7 Correlation analysis between CoDEGs and immune cells

Package “xCell”, based on a spillover compensation technique, was imported to infer 64 immune and stromal cell types. The Wilcoxon test was used to compare the differences between groups. Package “ggplot2” was used for the box plot of immune cells. The function “cor” in package “stats” was used to evaluate the correlation between coDEGs and immune cells. The correlation analysess were applied to up-regulated and down-regulated coDEGs respectively and the results were visualized by heatmap.

### 2.8 Identification of immune-cell-related CoDEGs

To identify the key genes that associated with immune cells among all coDEGs, WGCNA and differential analysis were considered. The following criteria were established to identify key genes: 1) The correlations with specific immune cells is more than 0.5 in both AS and IBD datasets. 2) Log foldchange (Log FC) > 0.3 in both AS and IBD datasets. The co-regulation of identified key genes was analyzed according to WGCNA.

### 2.9 Least absolute shrinkage and selection operator regression and receiver operating characteristic analysis

Least absolute shrinkage and selection operator (LASSO) regression were conducted to construct regression models. Up-regulated and down-regulated key genes were modeled separately. Receiver operating characteristic (ROC) analysis was performed to predict the diagnostic effectiveness of the models. The area under the ROC curve (AUC) value implied the diagnostic effectiveness in discriminating patients with AS or IBD from health controls in both AS and IBD datasets. Package “glmnet” was imported to perform the analyses.

### 2.10 Analysis of validation datasets

GSE25101 and GSE94648 were served as validation datasets for AS and IBD respectively. Correlation analysis between genes and immune cells was performed to confirm whether up-regulated and down-regulated genes are also significantly associated with specific immune cells in validation datasets. ROC analysis was applied to validation datasets in order to verify the diagnostic capability of the model again. GSE17755, a dataset of RA, was used for reverse validation to identify whether this correlation was also present in RA.

## 3 Results

### 3.1 Common differentially expressed genes in AS and IBD

**Figure 2:**
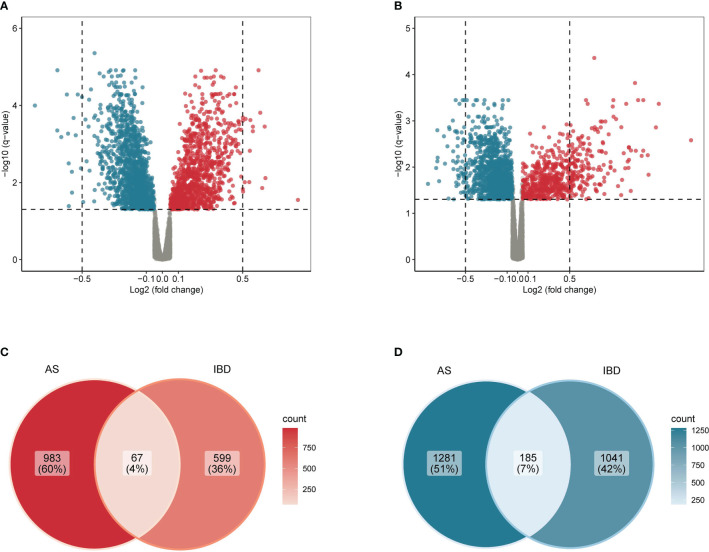
Common differentially expressed genes in AS and IBD **(A)** Volcano plot of differentially expressed genes in AS. **(B)** Volcano plot of differentially expressed genes in IBD. **(C)** Venn diagram of common up-regulated differentially expressed genes in AS and IBD. **(D)** Venn diagram of common down-regulated differentially expressed genes in AS and IBD.

A total of 141 samples were included in the analysis, including 52 patients and 20 health controls in GSE73754, as well as 45 patients with IBD and 24 health controls in GSE86434. Within GSE73754, 1050 up-regulated genes and 1466 down-regulated genes were identified between patients with AS and HC. Meanwhile, within GSE86434, 666 up-regulated genes and 1226 down-regulated genes were identified between patients with IBD and HC. Furthermore, 67 genes were up-regulated and 185 genes were down-regulated in both diseases. The results were presented in volcano plots and Venn diagrams (**Figure 2**). From another perspective, the co-up-regulated genes accounted for 6.4% of the total up-regulated genes in AS (67/1050) and 10.1% of that in IBD (67/666). Similarly, co-down-regulated genes accounted for 12.6% (185/1466) of the total down-regulated genes in AS and 15.1% (185/1226) in IBD.

### 3.2 PPI network construction and WGCNA

**Figure 3:**
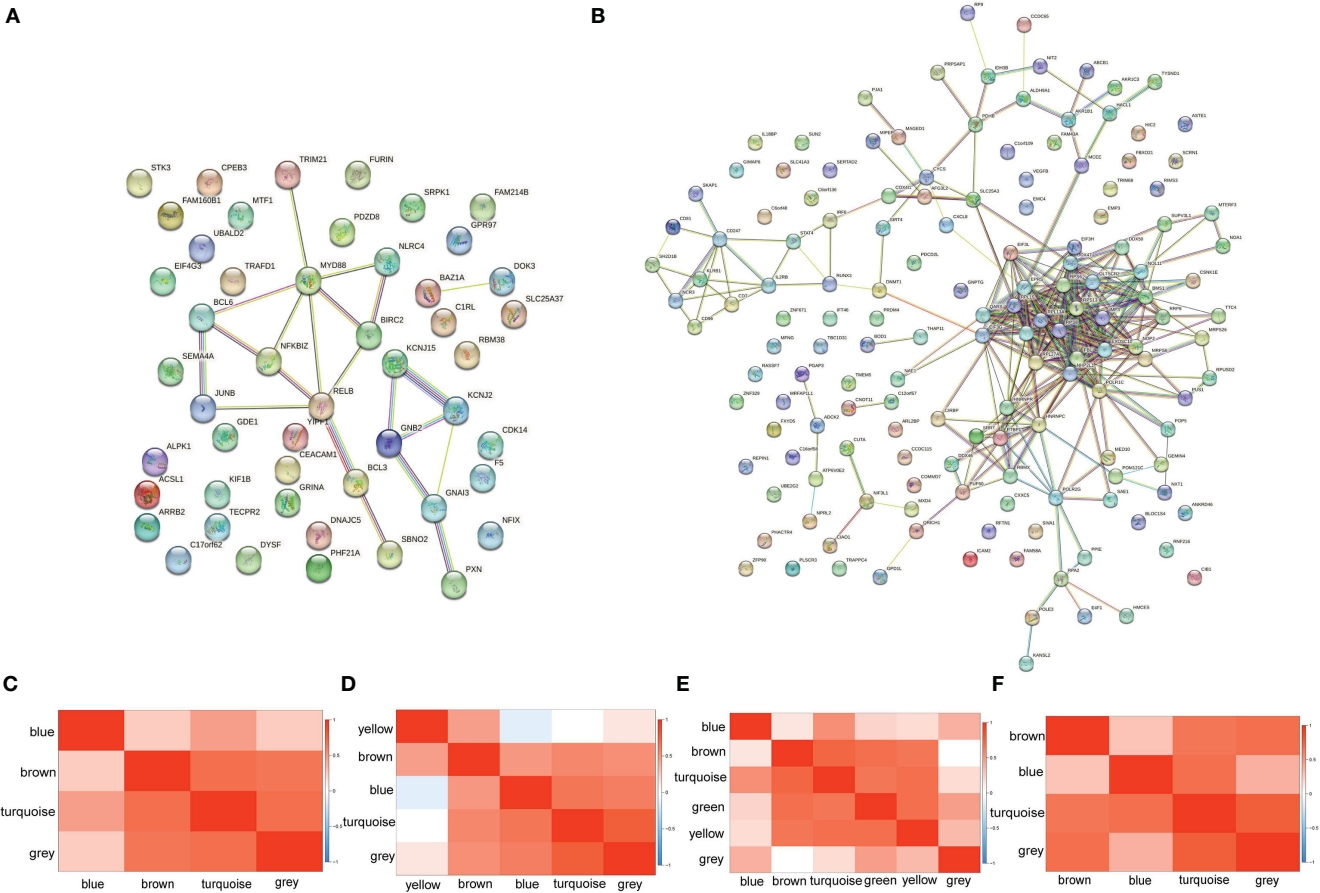
PPI network and WGCNA **(A)** PPI network of up-regulated coDEGs. **(B)** PPI network of down-regulated coDEGs. **(C)** Correlation between up-regulated gene modules in AS. **(D)** Correlation between down-regulated gene modules in AS. **(E)** Correlation between up-regulated gene modules in IBD. **(F)** Correlation between down-regulated gene modules in IBD.

PPI network and WGCNA were imported to explore how genes interact with each other. PPI networks of up-regulated genes and down-regulated genes were displayed in **Figures 3A, B**. Only 49 out of 67 up-regulated genes were performed since the function and properties of the remaining 18 genes have not yet been reported. Of the 49 genes, only 17 genes were included in the network, with MyD88 at its core. As for down-regulated coDEGs, only 153 out of 185 were performed, while 49 genes were isolated. FBL, with the highest centrality of 27, was the center of the network of down-regulated coDEGs, with the ability to methylate both RNAs and proteins. RPL4, NHP2L1, RPS8, IMP3, RPS6, RPS3, with centrality greater than 20, all associated with ribosome. WGCNA showed that up-regulated coDGEs could be divided into 4 modules in AS and 6 modules in IBD, while down-regulated coDEGs could be divided into 5 modules in AS and 4 modules in IBD (**Figures 3C–F**). Genes located in the same module in both AS and IBD were considered to be co-regulated in expression. Correlation analysis between modules showed that the overall correlation between modules deserved recognition. However, in WGCNA of AS, module yellow, consisted of IL2RB, AKR1C3 and SH2D1B, was less relevant to other modules. Also, the relationship between this module and immune cells was not like the other modules.

### 3.3 Common significant differential pathways in AS and IBD

**Figure 4:**
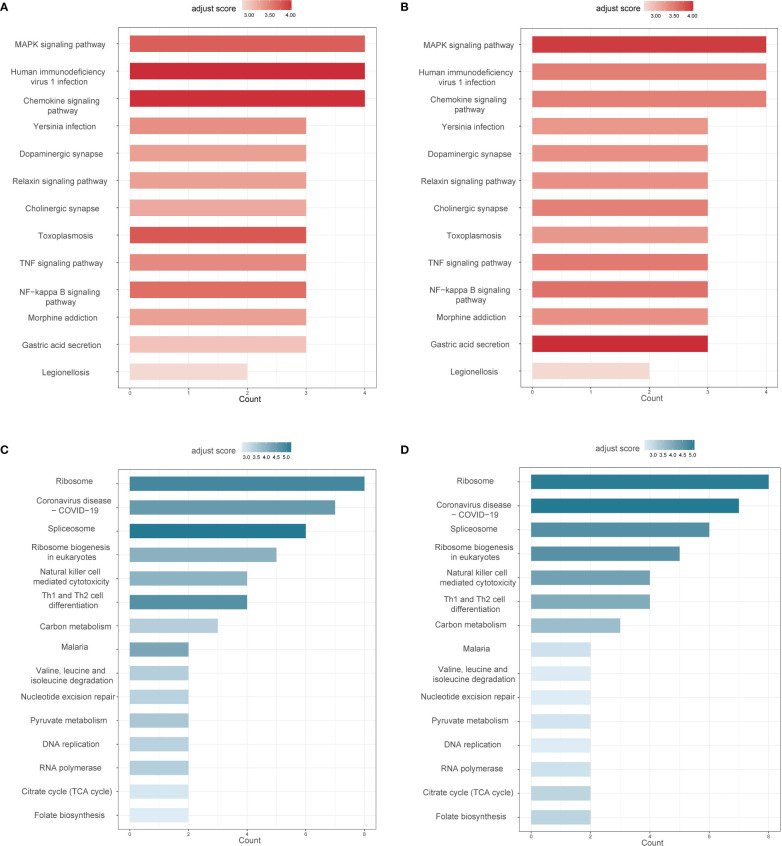
Common differential pathways in AS and IBD (adjust score calculated based on inverse cumulative distribution function) **(A)** Up-regulated differential pathways in AS. **(B)** Up-regulated differential pathways in IBD. **(C)** Down-regulated differential pathways in AS. **(D)** Down-regulated differential pathways in IBD.

**Figure 4** showed the up-regulated and down-regulated pathways in AS and IBD, respectively. Results suggested that the enrichment of down-regulated coDEGs was the highest in “Ribosome”, while “Spliceosome” also revealed its importance with count of 6. Both adjusted scores were rated highly, indicating their significant deviation from normal. Intriguingly, “Th1 and Th2 cell differentiation” and “Natural killer cell mediated cytotoxicity” were considered to be down-regulated in both AS and IBD according to the results, which might imply that helper T cells and NK cells were inhibited during the pathogenesis. This was also confirmed by the analysis of immune cells in the following.

“MAPK signaling pathway” and “Chemokine signaling pathway” were considered the most significant in up-regulated coDEGs enrichment analysis with both gene counts of 4 and high adjust scores. The results suggested that these two pathways not only enriched coDEGs, but also remarkably deviated from normal. “TNF signaling pathway” and “NF-κB signaling pathway” were also considered important, based on the analysis results and their critical position in inflammation.

**Figure 5:**
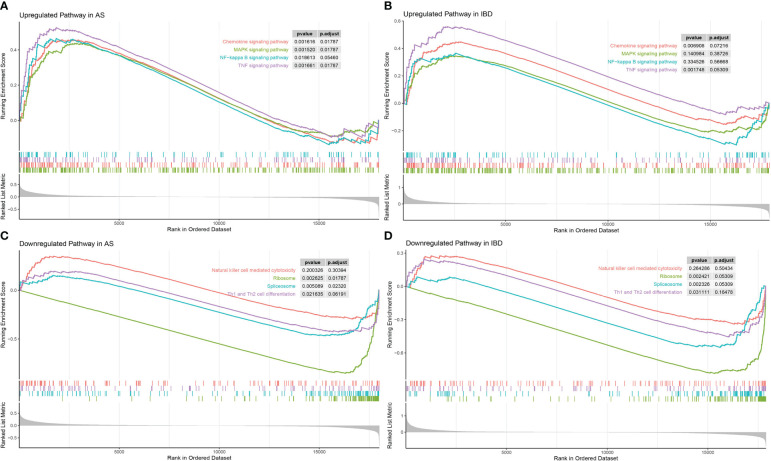
GSEA of significant common differential pathways in AS and IBD **(A)** GSEA of “MAPK signaling pathway”, “Chemokine signaling pathway”, “TNF signaling pathway”, “NF-κB signaling pathway” in AS. **(B)** GSEA of “MAPK signaling pathway”, “Chemokine signaling pathway”, “TNF signaling pathway”, “NF-κB signaling pathway” in IBD. **(C)** GSEA of “Ribosome”, “Spliceosome”, “Th1 and Th2 cell differentiation”, “Natural killer cell mediated cytotoxicity” in AS. **(D)** GSEA of “Ribosome”, “Spliceosome”, “Th1 and Th2 cell differentiation”, “Natural killer cell mediated cytotoxicity” in IBD.

Considering that the KEGG enrichment analysis was based on coDEGs, further confirmation of whether these pathways were significant in the overall biological state was requisite. GSEA was applied to “Ribosome”, “Spliceosome”, “Th1 and Th2 cell differentiation”, “Natural killer cell mediated cytotoxicity”, “MAPK signaling pathway”, “Chemokine signaling pathway”, “TNF signaling pathway” and “NF-κB signaling pathway” and the results were displayed in **Figure 5**. Among down-regulated pathways, “Ribosome”, “Spliceosome” and “Th1 and Th2 cell differentiation” were considered significant with P value < 0.05. Among up-regulated pathways, “TNF signaling pathway”, “MAPK signaling pathway” and “Chemokine signaling pathway” were significant in AS. However, “MAPK signaling pathway” was not significant in IBD, though the difference of “MAPK signaling pathway” was more obvious in KEGG analysis based on coDEGs. Meanwhile, “NF-κB signaling pathway” did not show significant differences in GSEA analysis.

### 3.4 Correlation Analysis between CoDEGs and immune cells

**Figure 6:**
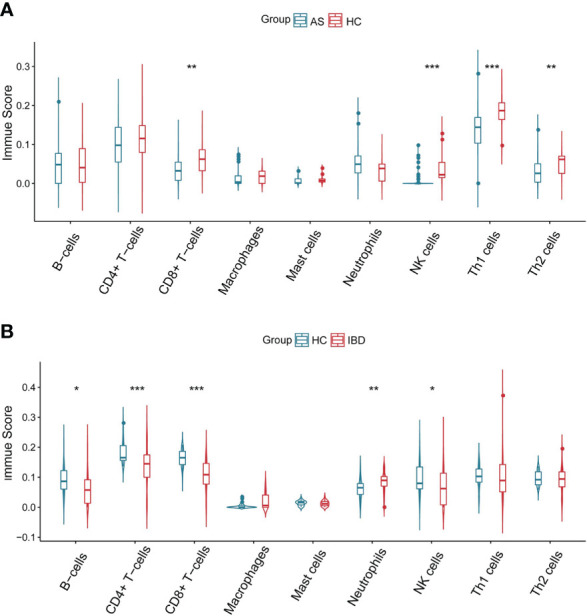
Immune scores of immune cells **(A)** Immune scores of immune cells in AS. **(B)** Immune scores of immune cells in IBD. *P<0.05 **P<0.01, ***P<0.001.

**Figure 6** showed differences in the expression of important immune cells between AS and IBD. Data from immune cell analysis revealed that CD8+ T−cells, Th1 cells, Th2 cells and NK cells were significantly lower in patients with AS compared to health controls, while CD8+ T−cells, CD4+ T−cells, B−cells and NK cells were significantly lower in patients with IBD. Only neutrophils performed a considerable upward trend in patients with IBD.

**Figure 7:**
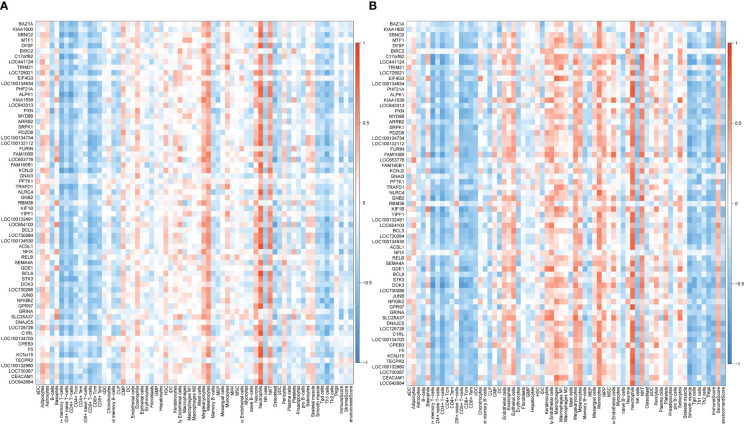
Correlation between up-regulated coDEGs and immune cells **(A)** Correlation between up-regulated coDEGs and immune cells in AS. **(B)** Correlation between up-regulated coDEGs and immune cells in IBD.

**Figure 8:**
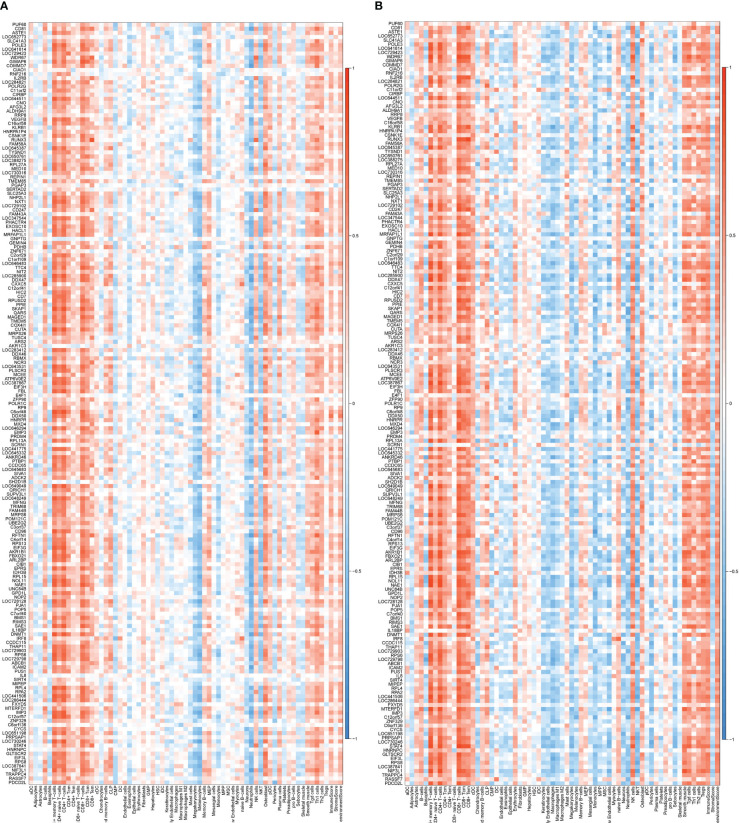
Correlation between down-regulated coDEGs and immune cells **(A)** Correlation between down-regulated coDEGs and immune cells in AS. **(B)** Correlation between down-regulated coDEGs and immune cells in IBD.

The correlation between coDEGs and immune cells was further analyzed. The results of up-regulated and down-regulated genes were shown in **Figures 7**, **8**, respectively. A clear trend is that up-regulated coDEGs were firmly associated with neutrophils, while down-regulated genes are significantly associated with CD8+ T−cells and CD4+ T−cells in both AS and IBD datasets.

### 3.5 Key immune-cell-related CoDEGs

The up-regulated gened associated immune cells were neutrophils, while the down-regulated gene associated cells were CD4+ and CD8+ T cells, which were the most significant cells in the correlation analysis. A total of 16 up-regulated genes and 20 down-regulated genes met the threshold for Log FC and correlation analysis. Seven of the 16 up-regulated genes and 14 of the 20 down-regulated genes, with names beginning with LOC, were excluded before the next analysis because they had not been studied. These genes were then identified by WGCNA to see if they belonged to the same module. Five out of 9 up-regulated genes and all 6 out of 6 down-regulated genes were observed in the same module in WGCNA of both AS and IBD. Finally, the up-regulated genes included in LASSO model were SBNO2, DYSF, SRPK1, ACSL1, BCL6, and the down-regulated genes were HNRPA1P4, C6orf48, ATP6V0E2, FBL, SKAP1, RPL13A. Complete key genes (before removing the unnamed genes) were presented in the supplemental materials.

### 3.6 LASSO regression model and ROC analysis

**Figure 9:**
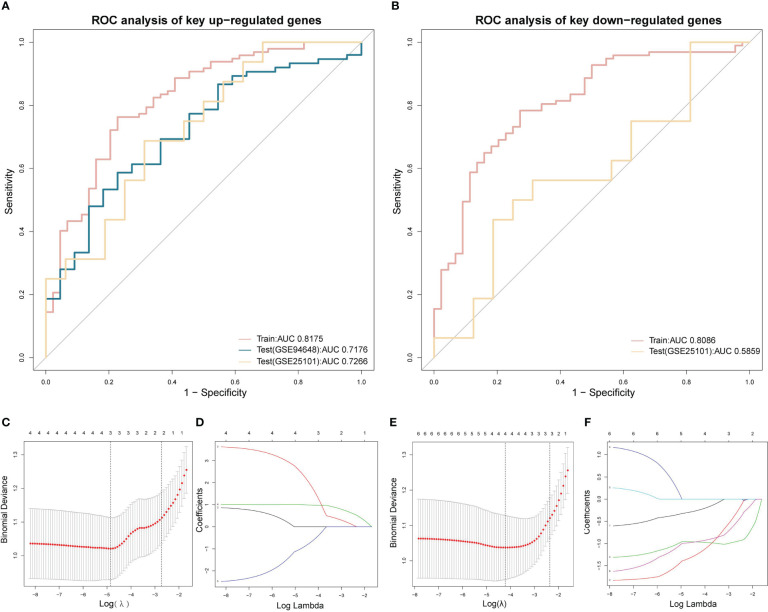
LASSO regression and ROC analysis **(A)** ROC of the LASSO regression model of up-regulated key coDEGs in train datasets (GSE73754 and GSE86434) and test datasets (GSE25101 and GSE94648). **(B)** ROC of the LASSO regression model of down-regulated key coDEGs in train datasets (GSE73754 and GSE86434) and test dataset (GSE25101). **(C)** Binomial deviance of LASSO regression model of up-regulated key coDEGs. **(D)** Coefficients of LASSO regression model of up-regulated key coDEGs. **(E)** Binomial deviance of LASSO regression model of down-regulated key coDEGs. **(F)** Coefficients of LASSO regression model of down-regulated key coDEGs.

The LASSO regression analysis of up-regulated key genes showed that SRPK1, ACSL1, and BCL6 were eventually included in the model. On the other hand, the model of down-regulated key genes finally consisted of HNRPA1P4, SKAP1, ATP6V0E2, and C6orf48. The selection of penalty term, λ, was displayed in **Figure 9**. ROC analyses suggested that both the up-regulated and down-regulated key gene models demonstrated a satisfactory ability to distinguish patients from the normal population with AUC of 0.82 and 0.81 respectively. Details of the final models were presented in supplemental materials.

### 3.7 Analysis of validation datasets

**Figure 10:**
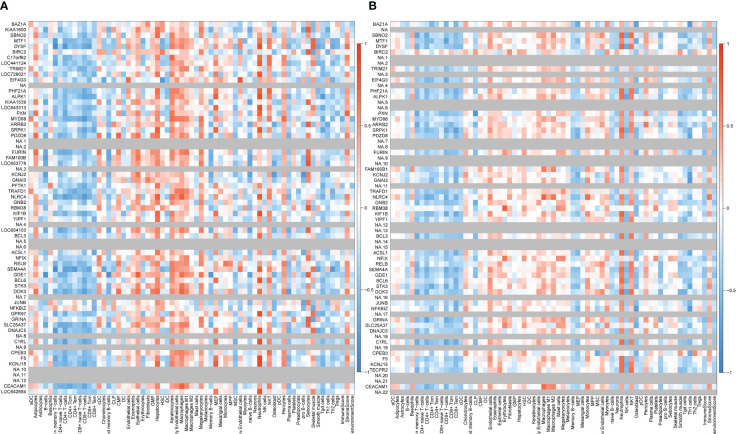
Correlation between up-regulated coDEGs and immune cells in validation datasets **(A)** Correlation between up-regulated coDEGs and immune cells in AS (GSE25101). **(B)** Correlation between up-regulated coDEGs and immune cells in IBD (GSE94648). (Gray means that the gene was not probed in this dataset).

**Figure 11:**
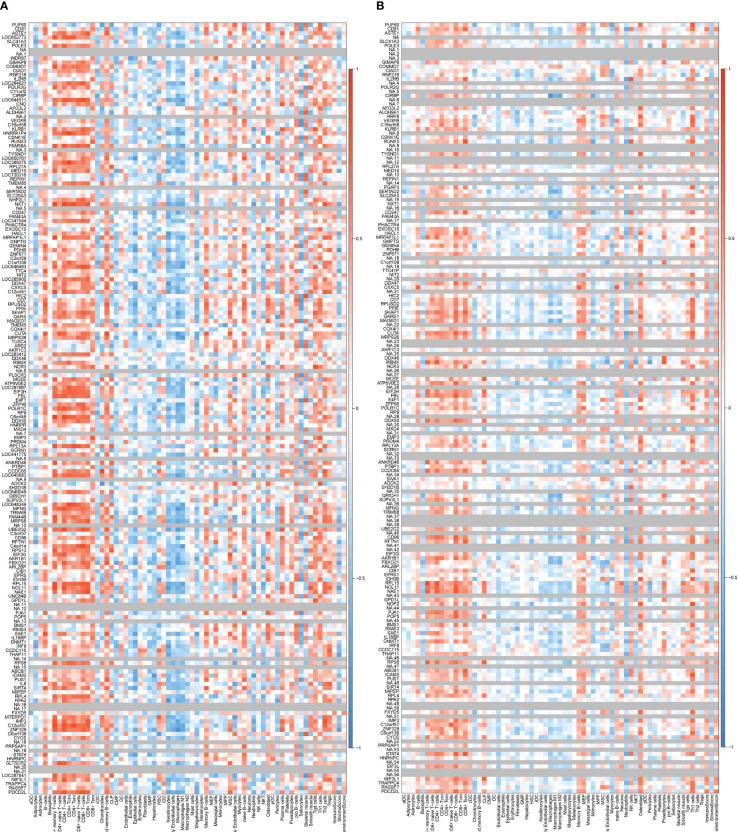
Correlation between down-regulated coDEGs and immune cells in validation datasets **(A)** Correlation between down-regulated coDEGs and immune cells in AS (GSE25101). **(B)** Correlation between down-regulated coDEGs and immune cells in IBD (GSE94648). (Gray means that the gene was not probed in this dataset.).

Two key results were verified using GSE25101 and GSE94648. On one hand, the validation datasets reconfirmed the correlation between up-regulated coDGEs and neutrophils, as well as the correlation between down-regulated coDGEs with CD8+ T−cells and CD4+ T−cells in both AS and IBD (**Figures 10**, **11**). It was interesting to note that associations with certain immune cells such as mast cells, Tregs and pDCs appeared differently in different datasets, which might suggest the effect of these cells may go both ways. On the other hand, the validation datasets were served as testing data to validate the performance of the two models in the additional AS and IBD datasets. The up-regulated key gene model performed better, with an AUC of 0.73 in the validation dataset of AS (GSE25101) and an AUC of 0.72 in the validation dataset of IBD (GSE94648) (**Figure 9A**). Due to the lack of HNRPA1P4 and C6orf48 expression data in GSE94648, the down-regulated gene model was only tested in GSE25101, with an AUC of 0.59 (**Figure 9B**). GSE94648 was tested with an incomplete model and the result was performed in **Supplementary Figure 3**.

**Figure 12:**
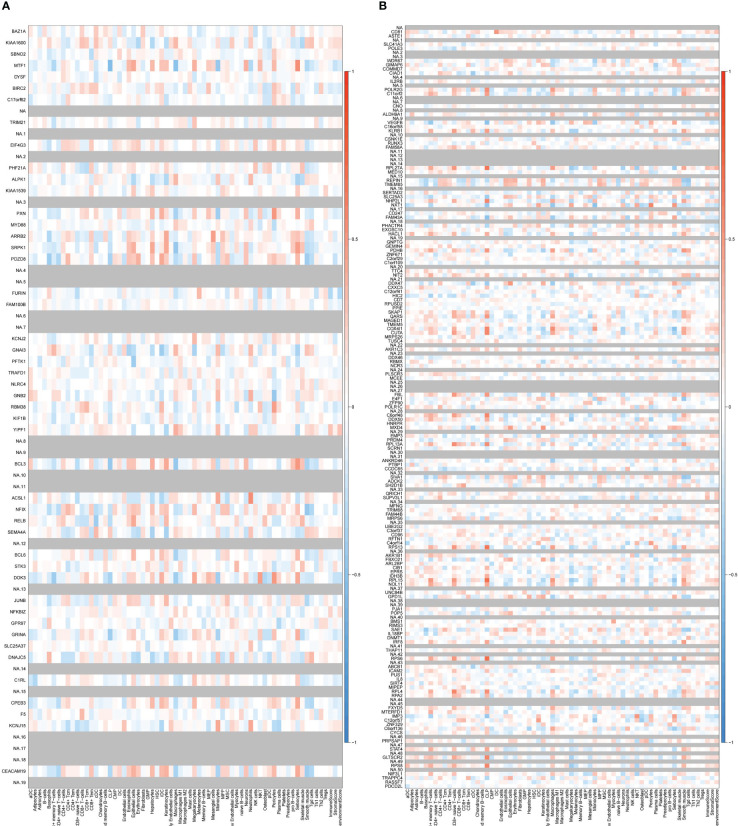
Correlation between up-regulated and down-regulated coDEGs and immune cells in RA datasets **(A)** Correlation between up-regulated coDEGs and immune cells in RA(GSE17755). **(B)** Correlation between down-regulated coDEGs and immune cells in RA (GSE17755). (Gray means that the gene was not probed in this dataset.).

Validation in GSE17755 showed that these genes did not exhibit a positive correlation with neutrophils or a negative correlation with CD4+ or CD8+ T cells in RA patients (**Figure 12**). This result suggested that the correlation was not widespread and further implied a special place for this correlation in the common pathogenesis of AS and IBD. P value and logFC of coDEGs in the validation datasets (GSE25101, GSE94648, GSE17755) were performed in **Supplementary Figures 1, 2**.

## 4 Discussion

The tight relationship between AS and IBD is rooted in their shared genetic background and pathogenesis, while the similarity in clinical manifestations is an external reflection. The current perspective is that barrier damage of mucosal surfaces provides an opportunity for subsequent exposure of the immune system to microorganisms (16), which may partly explain the considerably higher incidence of AS in IBD patients. The overlap between AS susceptibility loci and IBD loci makes this process more possible (17). The effectiveness of TNFi in treating both AS and IBD suggests that the two diseases are similar in the end immune response hierarchy (18, 19).

However, the exact common mechanism of the two diseases remains unclear, especially how local intestinal damage leads to systemic joint inflammation, mainly the axial. The peripheral blood is likely to act as a bridge in this process. Therefore, this study analyzed the gene expression in peripheral blood and focused on the common mechanism of AS and IBD, especially the involvement of immune cells.

### 4.1 How might genes play their roles?

The analysis showed that though the sheer number of the coDEGs were not large, the ratio was remarkable. However, the top 20 genes with the highest logFC in AS did not overlap with those in IBD, suggesting that the regulatory differences between the two diseases. The top five co-up-regulated genes with the highest logFC in both AS and IBD were DYSF, LOC653778, LOC654103, GRINA, and ACSL1.

Dysferlin, belonging to ferlin family, is a sarcolemmal protein that is implicated in calcium-dependent membrane repair (20). GRINA, belonging to the Lifeguard family, is involved in calcium homeostasis, which governs cell survival (21). ACSL1 is reported to be involved in fatty acid synthesis of triglyceride (22) and ferroptotic cell death triggered by conjugated linolenic acids (23). Also, ACSL1 could activate ATF6 signaling pathway and the inhibitors of ACSL1 significantly reduced the intestinal inflammation, the death of intestinal epithelial cells and the expression of TNF in Atg16l1^ΔIEC^ mice (24).

The top five co-down-regulated genes with the lowest logFC in both AS and IBD were KLRB1, IL2RB, IL8, LOC641814, and CD247. KLRB1 (CD161) is a C-type lectin-like receptor expressed on the majority of natural killer (NK) cells. Recently study found that the expression of CD161 was correlated with an innate responsiveness to cytokines in both T and NK cells and could identify NK cells that might contribute to inflammatory disease pathogenesis (25). Also it expressed on natural T (NT) cells and CD161+ T (NT) cells were reported to exist in human intestinal epithelium and might play an important role in local immunity (26). The amount of CD4+CD161+ T cells was observed significantly decreased in active ulcerative colitis (UC) compared with inactive UC (27). Since CD161 is expressed in a variety of cells, it remains to investigate which cells are involved in the decrease of CD161 expression in peripheral blood.

IL2RB is the β-chain of IL-2R, the receptor for IL-2 expressed on T cells and NK cells (28). The mutation of IL2RB could result in T and NK cell-driven immune dysregulation (29). A research suggested that an IL2RB single-nucleotide polymorphisms (SNPs) genotyped (rs743776) was significantly associated with Crohn’s disease, while IL2RA SNPs genotyped (rs4749924 and rs706778) were significantly associated with ulcerative colitis (30).

Interleukin-8 (IL-8) is a chemotactic cytokine for leukocytes, secreted by multiple cells, including monocytes and macrophages. It activates neutrophils and induces chemotaxis, causing migration of cells to the site of inflammation (31). Increased CXCL8 expression was observed in ulcerative colitis but data for Crohn’s disease is controversial (32). Also, IL-8 could be a predictor of relapse in IBD (33).

CD247, expressed primarily in natural killer (NK) and T cells, is component of TCR-CD3 complex composition and involved in the activation of T lymphocytes (34). So far, how does it participate in IBD and AS have not been studied.

To sum up, the role of these genes in AS and IBD is still very limited, especially the interactions between these genes and how they contribute to the pathogenesis of AS and IBD.

### 4.2 How might pathways be involved?

The results showed that “Ribosome”, “Spliceosome”, “Th1 and Th2 cell differentiation”, and “Natural killer cell mediated cytotoxicity” were the most significant down-regulated gene related pathways.

The ribosome, comprising two ribonucleoprotein subunits, is responsible for protein translation and synthesis (35). Significant down-regulation of ribosome-related genes was observed, including MRPS6, RPL13A, RPL15, RPL27A, RPL4, RPS13, RPS6, and RPS8. The heterogeneity in ribosomes may lead to a preference for the translation of particular mRNAs (36).

Spliceosome, a multimegadalton ribonucleoprotein (RNP) complex comprised of five snRNPs and numerous proteins, specializes in pre-mRNA splicing (37). In the study, the associated DDX46, HNRNPC, PPIE, PUF60, RBMX, and RP9 were found to be down-regulated. This provides a guess as to whether the down-regulation of the expression of these genes, related to ribosome and splicesome, may affect the transcription and translation of certain proteins in AS and IBD.

The genes of interest were CD247, ICAM2, NCR3 and SH2D1B in pathway “Natural killer cell mediated cytotoxicity” and CD247, IL2RB, RUNX3 and STAT4 in pathway “Th1 and Th2 cell differentiation”. Significant down-regulation of cell surface receptors in NK cells (CD247, NCR3) and T cells (CD247, IL2RB) is a feature. CD247 and IL2RB, mentioned above, are expressed both in NK cells and T cells. In a CD247-deficient patient, the natural killer cell was reported to be hyporesponsiveness and impaired development (38). The natural cytotoxicity receptor-3 (NCR3) is closely related to the activation of NK cells and its splice variants orchestrate the distinct functions of NK cell subtypes (39). The reduced expression of NCR3 was observed with reduced cytotoxicity and interferon-γ (IFN-γ) secretion in NK cells (40). Unlike surface receptors, STAT4 is located in cytoplasm and activates gene transcription in response to a variety of cytokines *via* the Janus kinase (JAK)-STAT signaling pathway (41). A study showed that the hypofunctionality of NK cells was associated with impaired STAT4 phosphorylation and down-regulation of the STAT4 target microRNA-155 (40). Another research indicated that IL-2 induced tyrosine phosphorylation of STAT4 in NK cells (42). Given that both STAT4 and IL-2RB were down-regulated in this study, IL-2/JAK/STAT pathway could be a possible mechanism. STAT4 activated by LIF (leukemia inhibitory factor) was found to inhibit Th17 accumulation and promote repair of damaged intestinal epithelium (43), demonstrating its ability to regulate T cells in IBD. RUNX3, like STAT4, belongs to transcription factors and served as a prominent regulator of multiple hematopoietic cell lineages, including CD8+ T cells, T helpers, natural killers, and B cells (43). According to a systematic review, the dysregulation of RUNX3, mostly in the form of deficiency, likely contributed to IBD pathogenesis (44).

The significant pathways of down-regulated genes performed two important aspects. On one hand, the transcription and translation of proteins, which are mainly completed by ribosomes and spliceosomes, may be affected. On the other hand, the activation of NK cells and T cells may be inhibited, which may be related not only to surface receptors but also to intracellular signal transduction.

Next part is about up-regulated coDEGs related pathways, of which “Chemokine signaling pathway”, “MAPK signaling pathway”, “TNF signaling pathway” and “NF-κB signaling pathway” are of interest.

In “Chemokine signaling pathway”, the study found that GNAI3 and GNB2, the subunit of guanine nucleotide-binding proteins (G proteins), were significantly up-regulated. On the contrary, β-arrestin 2, binding to the GPRK-phosphorylated receptor and precluding its coupling to the cognate G-protein, was also up-regulated. Meanwhile, in “MAPK signaling pathway”, the up-regulation of MYD88, a canonical adaptor located downstream of the Toll-like receptor (TLR) and interleukin-1 (IL-1) receptor families (45), was observed in the study. However, the expression of downstream gene did not show significant difference in the two pathways.

In the related research field of AS and IBD, most studies focused on MYD88, while G protein was relatively rare. Many therapies against IBD have been shown to act through MYD88-dependent signal pathways, mostly TLR4/MYD88 pathway (46–49), implying its prominent role in the pathogenesis. As for the G protein-related genes, one study reported that GNAI3 reduced colitis-associated tumorigenesis in mice (50). However, it remains to be elucidated why the downstream related gene expression is not significantly abnormal, which may hint at some unknown regulatory mechanism.

In “TNF signaling pathway”, BCL3, BIRC2 and JUNB were found up-regulated, but they were not the main signal transduction molecules in the pathway. Meanwhile, BIRC2, MYD88 and RELB were up-regulated in “NF-κB signaling pathway”, in which RELB, a member of the NF-κB family, could have important implication. These two pathways are actively involved in the pathogenesis of IBD and AS, however, how these genes regulate the pathways is still to be explored.

### 4.3 How can immune cells take part?

The most striking trend among the up-regulated genes was the correlation with neutrophils. Neutrophils, as the most abundant type of granulocytes in the immune system, can respond to pathogenic agents by degranulation, production of reactive oxygen species (ROS), phagocytosis, and formation of neutrophil extracellular traps (NET) (51). Besides, some studies showed that neutrophils were able to secrete a variety of cytokines like IL-6, IL-10 and IL-17. Different phenotypes of neutrophils such as tumor-associated neutrophils (TANs) and low-density neutrophils (LDN) were reported, suggesting the existence of neutrophil heterogeneity (52). In addition to its classical position in innate immunity, the ability to regulate adaptive immunity has received increasing attention. Cytokines, including autocrine and exogenous, and the contact between neutrophils and T cells could induce the expression of MHC-II and costimulatory molecules on neutrophils, suggesting that neutrophils could play as antigen-presenting cells (APCs) in presenting antigens and activating T cells (53).

There have also been some clues as to how neutrophils act in AS and IBD. To date, several studies demonstrated that the presence of NETs was increased in IBD (54). In UC lamina propria mononuclear cells, NETs activated ERK1/2, then enhancing TNF-α and interleukin-1β production (55). Also, NET exacerbated intestinal damage, as well as triggered exposed phosphatidylserine (PS)-positive microparticle release and PS exposure on platelets and endothelial cells partially through TLR2 and TLR4, driving thrombotic tendency in active IBD (56). Except for NET, cytokines produced by neutrophils also greatly influence IBD. A study discovered that tissue-infiltrating CXCR1(+)CXCR2(+) neutrophils were the main producer of IL-23 in the colon of paediatric patients with IBD, which might involved in disease initiation and progression (57).

In addition to the function of neutrophils themselves, they also affect IBD through their interaction with T cells. Extravasated neutrophils, isolated from the inflamed colon, were observed with enhanced surface expression of MHC class II and CD86, which might induce T cell activation and proliferation in IBD (58). Cathelicidin, found in the secondary granules of neutrophils and in NETs, is another potential factor. It was demonstrated that cathelicidin directed T cells from a Th1 to a Th17 phenotype and even protected Th17 from apoptosis in mouse lymph nodes (59). Considering that the expression of cathelicidin was up-regulated in the inflamed mucosa of IBD patients (60), this abnormality might further mediate changes in T cells.

Intriguingly, the role of neutrophils in IBD is bidirectional, that is, in addition to aggravating the disease, neutrophils have also been found to have a protective role (61). CD177+ neutrophils were notably increased in both peripheral blood and inflamed mucosa from patients with active IBD, while they exhibited a protective function (62). Spink7, mainly derived from neutrophils, showed a protective role in the murine colitis model, while Spink7-deficient mice preformed remarkably susceptible to experimental colitis (63).

The study of how neutrophils participate in the pathogenesis of AS is hitherto lacking. A recent research reported that neutrophils with inducible IL-23 production were observed in uninflamed human entheseal sites (64).

In contrast to neutrophils, CD4+ and CD8+ T cells were extremely associated with down-regulated genes. CD4+ T cells, known primarily as helper cells, not only own the ability to activate other immune cells like CD8+ T cells and B cells, but also display cytotoxic capabilities, suggesting a direct involvement in pathogen clearance. They are able to differentiate into several types, including T helper 1 (Th1), T helper 2 (Th2), T helper 17 (Th17), T helper 22 (Th22), regulatory T (Treg), and T follicular helper (Tfh) (65). Th17 cells promote tissue inflammation, while Tregs suppress autoimmunity in IBD. Also the imbalance between Th17 and Treg cells was observed (66). Tregs were decreased in the peripheral blood of UC mice model (67), which was consistent with the result in this study. Notably, Foxp3 expressing CD4+ T cells, belong to Treg, were also able to secret IL-17 in Crohn’s disease (68, 69), indicating a crossover between Th17 and Treg. Besides Th17, Th1 cells were also considered to be predominant gut-infiltrating proinflammatory T cell populations in IBD, expressing the transcription factor T-bet and the cytokine IFN-γ (70). Th2 cells, producing IL-4, IL-5, IL-13, were found to contribute to the pathogenesis of IBD (71). However, another study showed that Th2 cytokines rebalanced T-helper cell subsets and performed protective effect against colitis in BLIMP-1 knockout mice (72). As for Th22 cells, secreting IL-22, a study observed significantly reduced numbers of IL-22+ cells in actively inflamed tissues in UC patients (73).T follicular helper (Tfh) cells are specialized in helping B cells in the germinal center reaction. Research showed that IRF8-regulated Tfh could function as B-cell-independent, pathogenic, mediators of colitis (74).

Just as in IBD, CD4+ T cells also play a pivotal role in the pathogenesis of AS. A meta analysis showed that the proportions of CD4+ T cells, Th17 cells, Tfh cells as well as Th1/Th2 ratio were significantly increased and Tregs were significantly decreased among the subsets of T cells, while the percentages of T cells were not significantly different (75). The function of these CD4+T cells in the pathogenesis of AS seems to be similar to that in IBD. For example, Th17 promoted inflammation through the secretion of IL-17 (76). AS for Tregs, though the data on whether there was a decrease in the number of Tregs were controversial in AS patients, the function of Tregs appeared to be impaired (77).

The functions of CD8+T cells in IBD were reported controversial, with some reports indicating anti-colitogenic properties and others showing their contribution to tissue inflammation, which could be explained by the diversity of sources and subtypes (78).The best known CD8+ T cells, also called cytotoxic T lymphocytes (CTL), are characterized by robust production of IFN-γ and cytolytic activities (79). The increase of activated CTLs was observed both in peripheral blood and the affected intestinal mucosa (80, 81). In addition, animal models showed that antigen-specific CD8+ T cells could induce relapsing colitis (82). Meanwhile, the cytokines secreted by the CTLs, like type I IFN, were causative factors of intestinal leakage (83). These studies support the involvement of CTLs in the pathogenesis of IBD.

In contrast to the effect of CTLs, CD8+ regulatory T cells are able to effectively block the overreacting immune responses and maintain the body’s immune homeostasis. A research reported that UC was associated with increased colonic regulatory T cells characterized by elevated expression of ZEB2 (84). However, the defects in CD8+ regulatory T cells in the lamina propria of IBD patients were observed, as shown by an inability to suppress immune globulin production by peripheral blood mononuclear cells (PBMC) (85).

Except for CTLs and CD8+ Tregs, a type of IL-17-producing CD8+ T cells (Tc17) also participate in the formation of colitis (86). The abnormal increase of Tc17 in peripheral blood and tissues was demonstrated (87).

With regard to AS, much evidence confirmed the involvement of CD8+ T cells in its pathogenesis, especially given the pivotal position of HLA-B27 in AS. A theory called “arthritogenic peptide” proposed that HLA-B27 presented peptides derived from exogenous sources to CD8+ T cells, which subsequently cross-reacted with antigens and causing inflammation (88). As early as 1993, B27-restricted CTLs with specificity for arthritogenic bacteria or autoantigens were observed in the synovial fluid of AS patients (89). Then several later studies identifyed specific TCR β motifs in AS (90, 91). These lines of evidence all suggest that CD8+ T cells may be deeply specific players in the pathogenesis of AS.

It is thought-provoking that our study showed that both CD4+ T cells and CD8+ T cells decreased, as inferred from gene expression, in IBD and AS patients. This did not coordinate with some of the studies mentioned above. One possible explanation is that there are many subsets of CD4+ T cells and CD8+ T cells, while some of them have increased and others have not. Actually, a study revealed a reduced size of T-cell expansions in AS patient blood (92).

### 4.4 How might CoDEGs regulate immune cells?

The study focused on genes that were highly variable in both AS and IBD (both logFc > 0.3) and were greatly associated with immune cells (both correlations > 0.5). Surprisingly, the proportion of undefined genes, with names beginning with LOC, was even higher than 50% in the final results (7 out of 16 in neutrophils-associated genes and 14 out of 20 in T-cells-associated genes). This may suggest that a large number of co-regulated genes whose roles remain to be explored.

How the five neutrophil-related genes, SBNO2, DYSF, SRPK1, ACSL1, and BCL6, act on neutrophils has not been systematically studied, but some hints suggest a potential link. In dysferlin (DYSF)-deficient mice, there was a reduction in early neutrophils recruitment in regenerating muscle (93). A review pointed to a possible role for ACSL1 in inflammasome activation in neutrophils (94). BCL6 deficiency was demonstrated to promote tissue neutrophil apoptosis, which could be due to the fact that BCL6 was able to bound to the neutrophil gene loci involved in cellular apoptosis in cells specifically at the site of infection and then BCL6 disruption led to increased expression of apoptotic genes in neutrophils (95). As for SBNO2 and SRPK1, there were no relevant studies on their association with neutrophils.

SKAP1, one of the six T-cell-related genes, was proved to be crucial in regulation of T cells while there was very limited research on how other 5 genes (ATP6V0E2, C6orf48, FBL, HNRPA1P4, RPL13A) influence T cells. N-SKAP1-C-RapL was an “inside-out” pathway that regulates T cell adhesion, motility, and arrest times with T cell-dendritic cells in lymph nodes (96). GRB-2-SKAP1, an adaptor complex, was involved in regulating the conjugation of T cells to dendritic cells (DC) (97). Also, SKAP1 was found to affect the function of talin in T-cells needed for optimal T-cell/DC conjugation (98). Although there were no studies of SKAP1 in AS, Skap1-deficient (skap1-/-) mice performed highly resistant to the induction of collagen-induced arthritis, both in terms of incidence or severity, which presumably because of a marked reduction of joint infiltrating T cells as well as a selective reduction in the presence of IL-17+ (Th17) in Skap1-/- T cells (99).

In general, relevant studies of these immune-cell-related genes identified in this study are fairly scarce. Not only are there a large number of genes that have not yet to be named, but also how the genes with partially known functions act on the regulation of immune cells also remains to be investigated.

### 4.5 Where has this study arrived and where to go next?

This study aimed to investigate the possible common pathogenesis of AS and IBD through the changes in gene expression in peripheral blood. With this aim, possible pathways were analyzed with up-regulated and down-regulated coDEGs. Furthermore, the involvement of immune cells was analyzed, and it came out that neutrophils were most associated with up-regulated coDEGs while CD4+ and CD8+ T cells were most associated with down-regulated coDEGs. Finally, the most relevant up-regulated coDEGs for neutrophils and the most relevant down-regulated coDEGs for T cells were revealed, and then diagnostic models were constructed, demonstrating the specificity of the co-regulation mode of these genes in AS and IBD.

A major limitation of this study was that the analyses were based on gene expression assays without cellular validation. To make our conclusions more robust, we introduced two validation datasets for AS and IBD, respectively. A consistent main result was observed in both validation datasets, namely a strong association of coDEGs with neutrophils and T cells. More importantly, the dataset of RA showed that this correlation was not ubiquitous, which reinforced the results of the study.

This study provides new insights into the co-pathogenesis of AS and IBD. It is proposed that neutrophils and T cells may be actively involved in this process, and the genes most relevant to their regulation are revealed in this paper, which has not previously been performed. In addition, a review of the possible mechanisms of how neutrophils and T cells and their related genes participate in the pathogenesis of AS and IBD are presented to provide a more complete view. However, there are still three important links that need to be connected: These genes, through what pathways, and then how to act on immune cells, and finally, how to affect the occurrence and process of disease?

## Data availability statement

The datasets presented in this study can be found in online repositories. The names of the repository/repositories and accession number(s) can be found in the article/**Supplementary Material**.

## Author contributions

YD was contributed to data collection, analysis and writing the original draft. YY and LX contributed to validation and project administration, resources, review, editing and supervision. All authors contributed to the article and approved the submitted version.
