# Supplementary material for: Immune cells and their related genes provide a new perspective on the common pathogenesis of ankylosing spondylitis and inflammatory bowel diseases
Source: Front Immunol. 2023 Mar 30;14:1137523. doi: 10.3389/fimmu.2023.1137523 (PMC10101339; doi:10.3389/fimmu.2023.1137523)
Supplement: Supplementary file 1 [file DataSheet_1.docx]

1. Key immue-cell-related coDEGs

Up-regulated key genes

KIAA1600

SBNO2

DYSF

LOC441124

LOC729021

LOC100134634

ALPK1

LOC643313

SRPK1

LOC100132112

KCNJ2

LOC730284

LOC100134530

ACSL1

BCL6

KCNJ15

Down-regulated key genes

LOC283412

LOC729102

HNRPA1P4

LOC285900

LOC441506

LOC649049

C6orf48

ATP6V0E2

LOC648249

FBL

SKAP1

LOC284821

LOC347544

LOC387867

LOC646483

LOC645387

RPL13A

LOC641814

LOC441775

LOC644511

1. Details of the final LASSO regression models

Up-regulated gene model

6 x 1 sparse Matrix of class "dgCMatrix"

1

(Intercept) -21.734883

SBNO2 .

DYSF .

SRPK1 2.592003

ACSL1 1.007507

BCL6 -1.049651

Down-regulated gene model

7 x 1 sparse Matrix of class "dgCMatrix"

1

(Intercept) 33.9460471

HNRPA1P4 -0.2406054

SKAP1 -1.2062921

ATP6V0E2 -0.9683707

FBL .

RPL13A .

C6orf48 -0.9488224

1. The performance of coDEGs in the validation datasets (GSE25101, GSE94648, GSE17755)


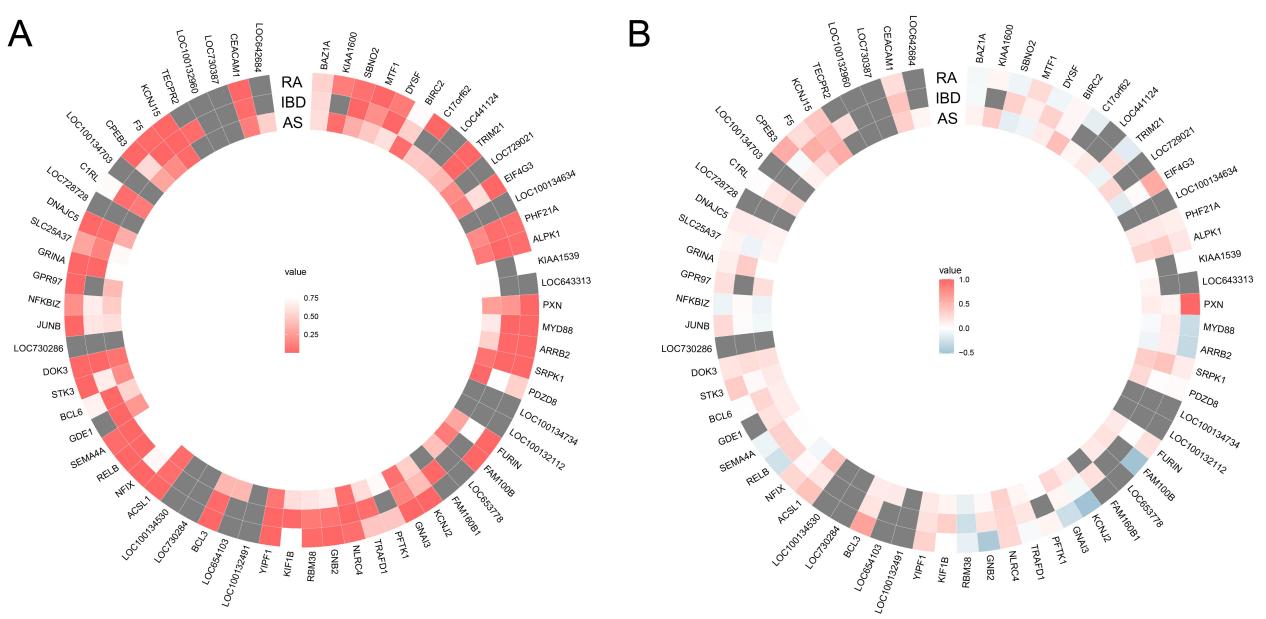


Supplementary Figure 1 P value and logFC of up-regulated coDEGs in the validation datasets of RA (GSE17755), IBD (GSE94648) and AS (GSE25101). (A) P value of up-regulated coDEGs in the validation datasets of RA, IBD and AS. (A) LogFC of up-regulated coDEGs in the validation datasets of RA, IBD and AS.


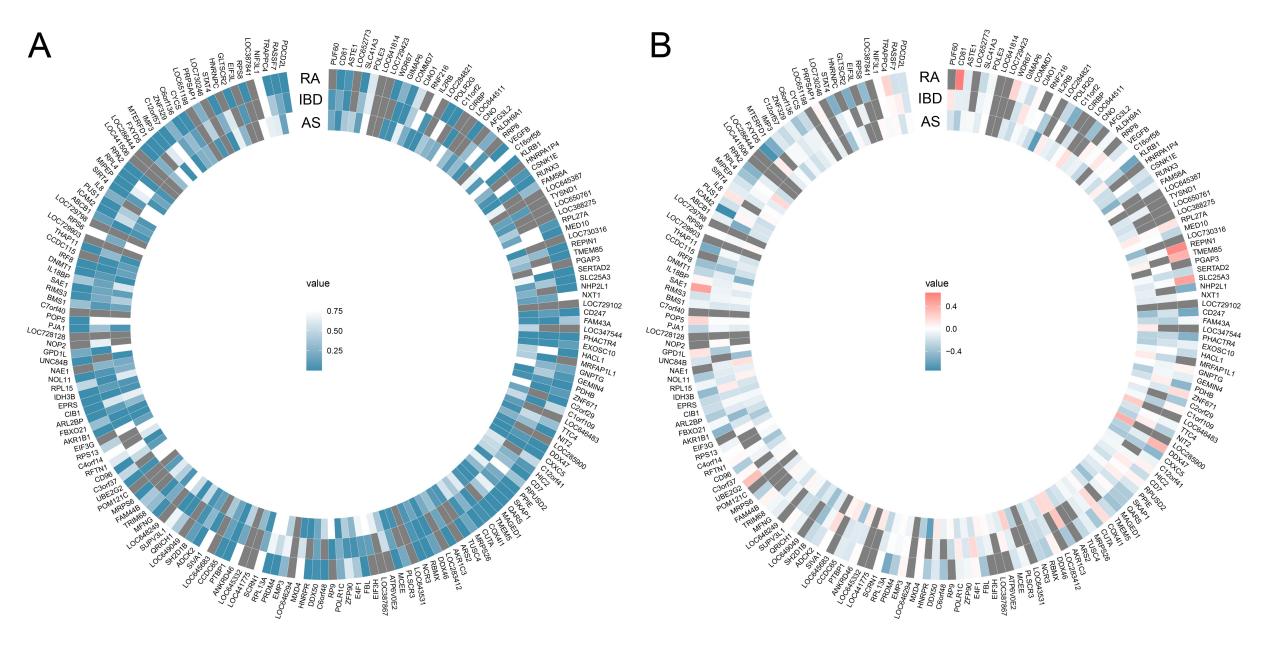


Supplementary Figure 2 P value and logFC of down-regulated coDEGs in the validation datasets of RA (GSE17755), IBD (GSE94648) and AS (GSE25101). (A) P value of down-regulated coDEGs in the validation datasets of RA, IBD and AS. (A) LogFC of down-regulated coDEGs in the validation datasets of RA, IBD and AS.

1. ROC of incomplete LASSO regression model of down-regulated key coDEGs in GSE94648


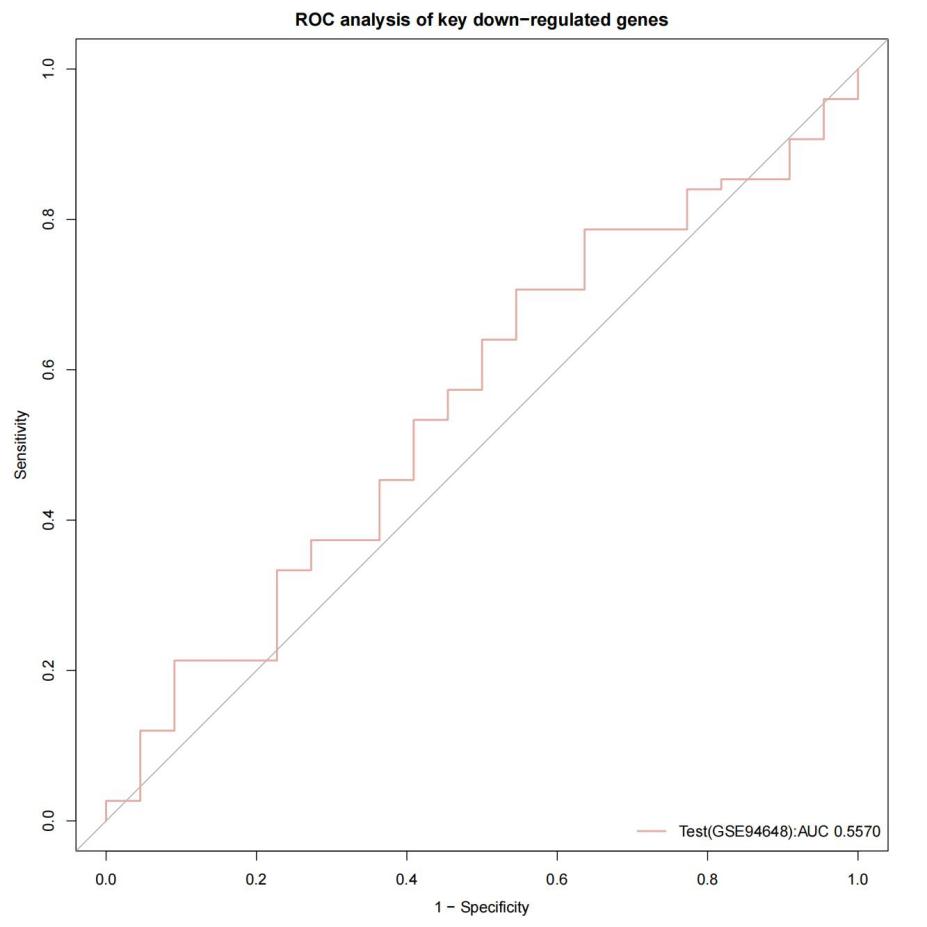


Supplementary Figure 3 ROC of the incomplete LASSO regression model of down-regulated key coDEGs (SKAP1 and ATP6V0E2) in GSE94648.
